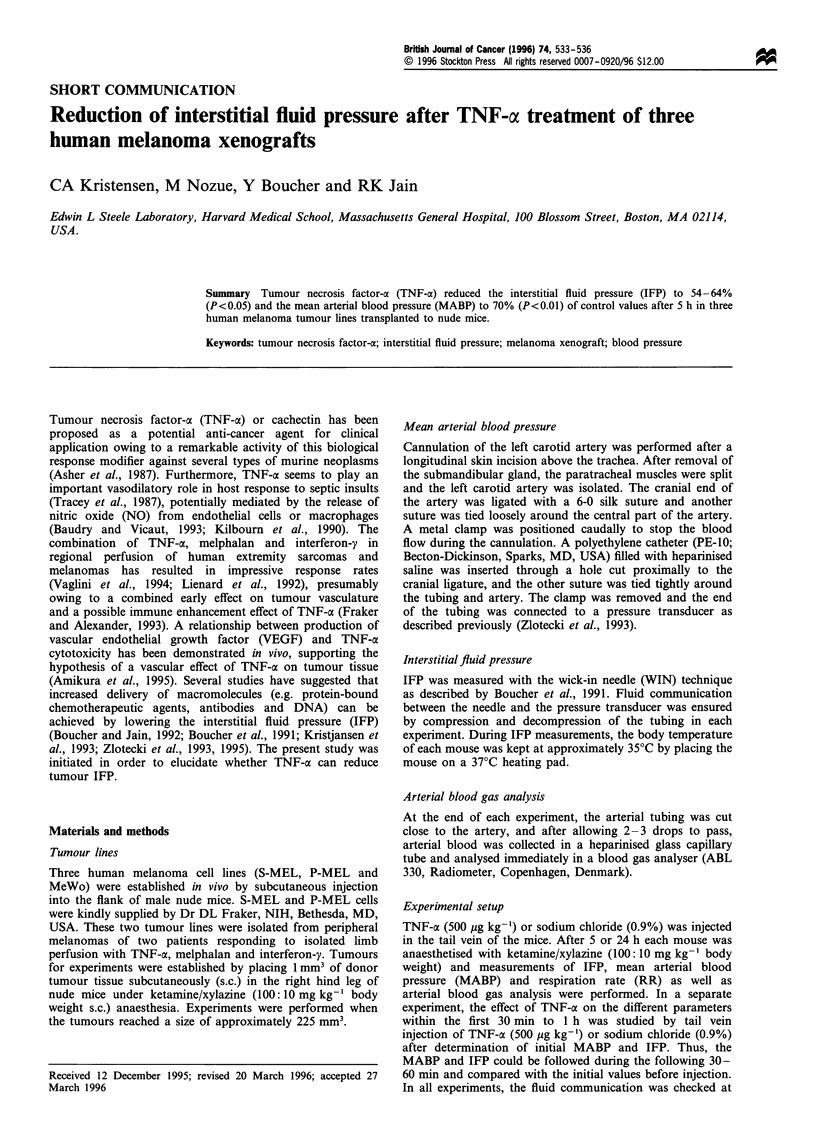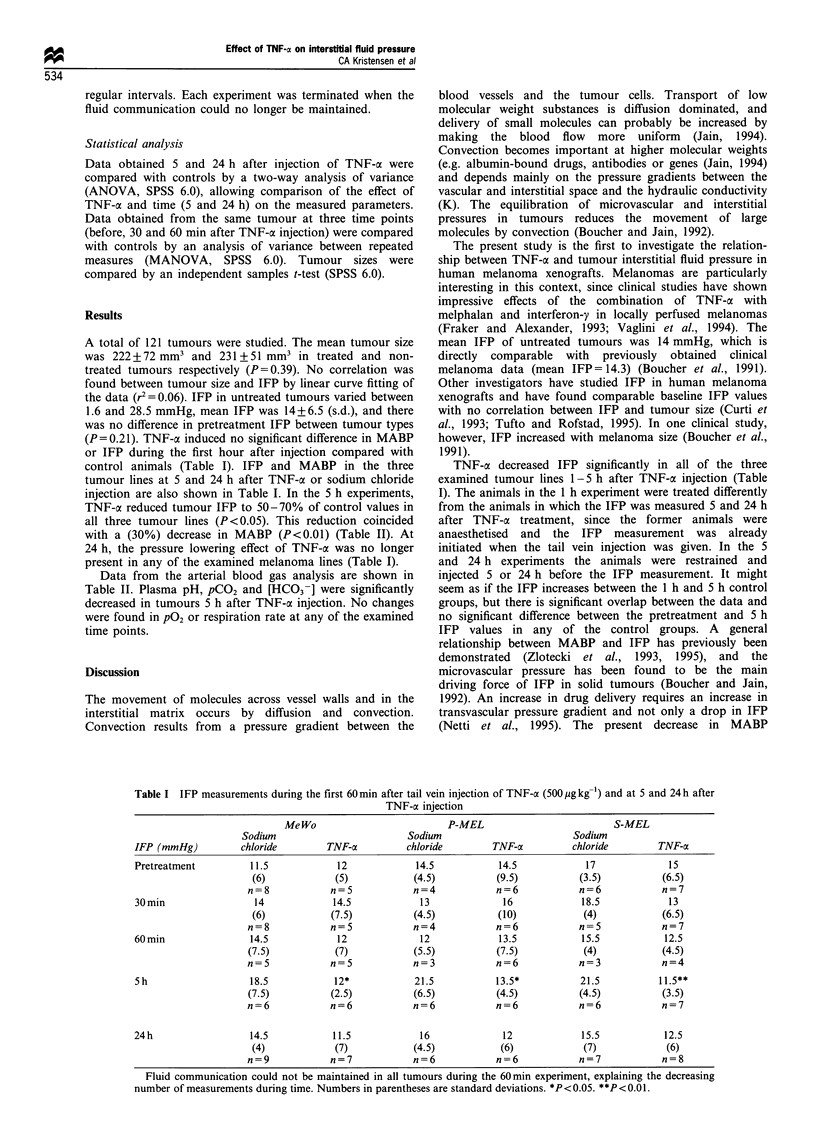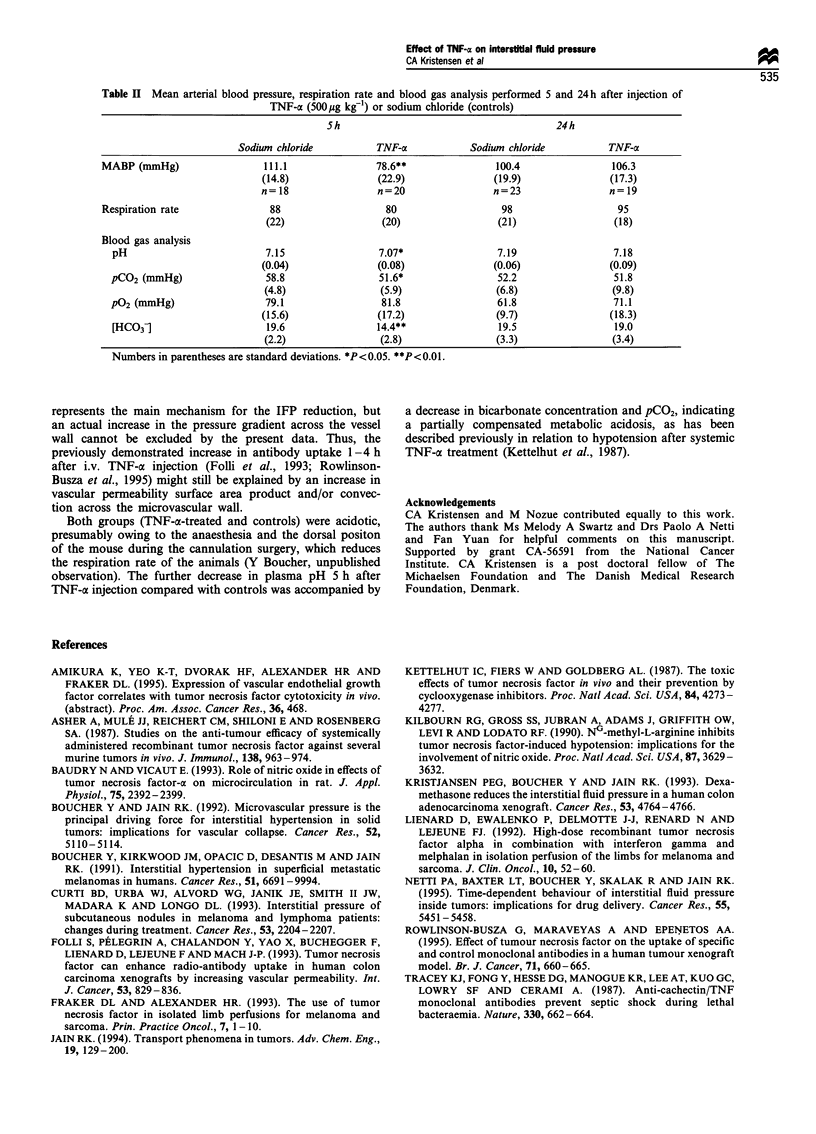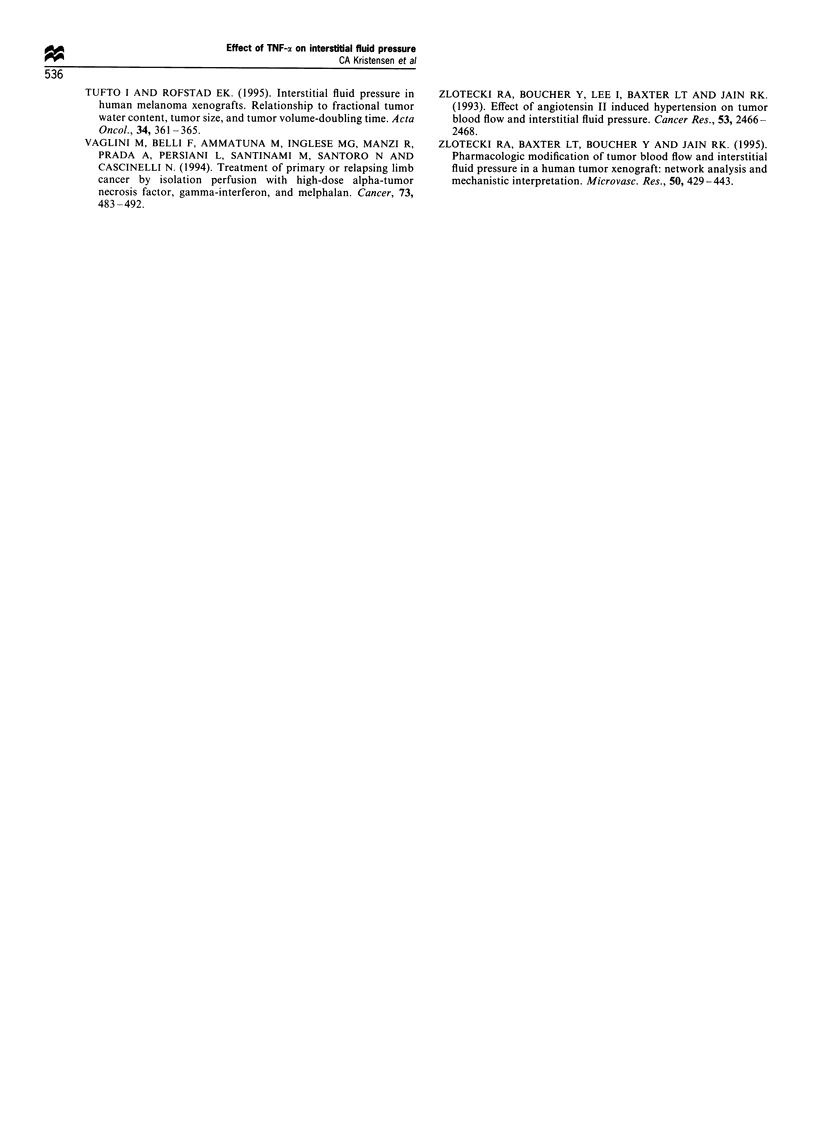# Reduction of interstitial fluid pressure after TNF-alpha treatment of three human melanoma xenografts.

**DOI:** 10.1038/bjc.1996.397

**Published:** 1996-08

**Authors:** C. A. Kristensen, M. Nozue, Y. Boucher, R. K. Jain

**Affiliations:** Edwin L Steele Laboratory, Harvard Medical School, Massachusetts General Hospital, Boston 02114, USA.

## Abstract

Tumour necrosis factor-alpha (TNF-alpha) reduced the interstitial fluid pressure (IFP) to 54-64% (P < 0.05) and the mean arterial blood pressure (MABP) to 70% (P < 0.01) of control values after 5 h in three human melanoma tumour lines transplanted to nude mice.


British Journal of Cancer (1996) 74, 533-536

© 1996 Stockton Press All rights reserved 0007-0920/96 $12.00

SHORT COMMUNICATION

### Reduction of interstitial fluid pressure after TNF-$\alpha$ treatment of three human melanoma xenografts

CA Kristensen, M Nozue, Y Boucher and RK Jain

*Edwin L Steele Laboratory, Harvard Medical School, Massachusetts General Hospital, 100 Blossom Street, Boston, MA 02114, USA.*

**Summary** Tumour necrosis factor-$\alpha$ (TNF-$\alpha$) reduced the interstitial fluid pressure (IFP) to 54-64% ($P<0.05$) and the mean arterial blood pressure (MABP) to 70% ($P<0.01$) of control values after 5 h in three human melanoma tumour lines transplanted to nude mice.

**Keywords:** tumour necrosis factor-$\alpha$; interstitial fluid pressure; melanoma xenograft; blood pressure

Tumour necrosis factor-$\alpha$ (TNF-$\alpha$) or cachectin has been proposed as a potential anti-cancer agent for clinical application owing to a remarkable activity of this biological response modifier against several types of murine neoplasms (Asher *et al.*, 1987). Furthermore, TNF-$\alpha$ seems to play an important vasodilatory role in host response to septic insults (Tracey *et al.*, 1987), potentially mediated by the release of nitric oxide (NO) from endothelial cells or macrophages (Baudry and Vicaut, 1993; Kilbourn *et al.*, 1990). The combination of TNF-$\alpha$, melphalan and interferon-$\gamma$ in regional perfusion of human extremity sarcomas and melanomas has resulted in impressive response rates (Vaglini *et al.*, 1994; Lienard *et al.*, 1992), presumably owing to a combined early effect on tumour vasculature and a possible immune enhancement effect of TNF-$\alpha$ (Fraker and Alexander, 1993). A relationship between production of vascular endothelial growth factor (VEGF) and TNF-$\alpha$ cytotoxicity has been demonstrated *in vivo*, supporting the hypothesis of a vascular effect of TNF-$\alpha$ on tumour tissue (Amikura *et al.*, 1995). Several studies have suggested that increased delivery of macromolecules (e.g. protein-bound chemotherapeutic agents, antibodies and DNA) can be achieved by lowering the interstitial fluid pressure (IFP) (Boucher and Jain, 1992; Boucher *et al.*, 1991; Kristjansen *et al.*, 1993; Zlotecki *et al.*, 1993, 1995). The present study was initiated in order to elucidate whether TNF-$\alpha$ can reduce tumour IFP.

### Materials and methods

### *Tumour lines*

Three human melanoma cell lines (S-MEL, P-MEL and MeWo) were established *in vivo* by subcutaneous injection into the flank of male nude mice. S-MEL and P-MEL cells were kindly supplied by Dr DL Fraker, NIH, Bethesda, MD, USA. These two tumour lines were isolated from peripheral melanomas of two patients responding to isolated limb perfusion with TNF-$\alpha$, melphalan and interferon-$\gamma$. Tumours for experiments were established by placing 1 $mm^3$ of donor tumour tissue subcutaneously (s.c.) in the right hind leg of nude mice under ketamine/xylazine (100:10 mg $kg^{-1}$ body weight s.c.) anaesthesia. Experiments were performed when the tumours reached a size of approximately 225 $mm^3$.

Received 12 December 1995; revised 20 March 1996; accepted 27 March 1996

### *Mean arterial blood pressure*

Cannulation of the left carotid artery was performed after a longitudinal skin incision above the trachea. After removal of the submandibular gland, the paratracheal muscles were split and the left carotid artery was isolated. The cranial end of the artery was ligated with a 6-0 silk suture and another suture was tied loosely around the central part of the artery. A metal clamp was positioned caudally to stop the blood flow during the cannulation. A polyethylene catheter (PE-10; Becton-Dickinson, Sparks, MD, USA) filled with heparinised saline was inserted through a hole cut proximally to the cranial ligature, and the other suture was tied tightly around the tubing and artery. The clamp was removed and the end of the tubing was connected to a pressure transducer as described previously (Zlotecki *et al.*, 1993).

### *Interstitial fluid pressure*

IFP was measured with the wick-in needle (WIN) technique as described by Boucher *et al.*, 1991. Fluid communication between the needle and the pressure transducer was ensured by compression and decompression of the tubing in each experiment. During IFP measurements, the body temperature of each mouse was kept at approximately 35°C by placing the mouse on a 37°C heating pad.

### *Arterial blood gas analysis*

At the end of each experiment, the arterial tubing was cut close to the artery, and after allowing 2-3 drops to pass, arterial blood was collected in a heparinised glass capillary tube and analysed immediately in a blood gas analyser (ABL 330, Radiometer, Copenhagen, Denmark).

### *Experimental setup*

TNF-$\alpha$ (500 $\mu g\ kg^{-1}$) or sodium chloride (0.9%) was injected in the tail vein of the mice. After 5 or 24 h each mouse was anaesthetised with ketamine/xylazine (100:10 mg $kg^{-1}$ body weight) and measurements of IFP, mean arterial blood pressure (MABP) and respiration rate (RR) as well as arterial blood gas analysis were performed. In a separate experiment, the effect of TNF-$\alpha$ on the different parameters within the first 30 min to 1 h was studied by tail vein injection of TNF-$\alpha$ (500 $\mu g\ kg^{-1}$) or sodium chloride (0.9%) after determination of initial MABP and IFP. Thus, the MABP and IFP could be followed during the following 30-60 min and compared with the initial values before injection. In all experiments, the fluid communication was checked at regular intervals. Each experiment was terminated when the fluid communication could no longer be maintained.

*Statistical analysis*

Data obtained 5 and 24 h after injection of TNF-α were compared with controls by a two-way analysis of variance (ANOVA, SPSS 6.0), allowing comparison of the effect of TNF-α and time (5 and 24 h) on the measured parameters. Data obtained from the same tumour at three time points (before, 30 and 60 min after TNF-α injection) were compared with controls by an analysis of variance between repeated measures (MANOVA, SPSS 6.0). Tumour sizes were compared by an independent samples *t*-test (SPSS 6.0).

### Results

A total of 121 tumours were studied. The mean tumour size was $222 \pm 72$ mm$^3$ and $231 \pm 51$ mm$^3$ in treated and non-treated tumours respectively ($P = 0.39$). No correlation was found between tumour size and IFP by linear curve fitting of the data ($r^2 = 0.06$). IFP in untreated tumours varied between 1.6 and 28.5 mmHg, mean IFP was $14 \pm 6.5$ (s.d.), and there was no difference in pretreatment IFP between tumour types ($P = 0.21$). TNF-α induced no significant difference in MABP or IFP during the first hour after injection compared with control animals (Table I). IFP and MABP in the three tumour lines at 5 and 24 h after TNF-α or sodium chloride injection are also shown in Table I. In the 5 h experiments, TNF-α reduced tumour IFP to 50–70% of control values in all three tumour lines ($P < 0.05$). This reduction coincided with a (30%) decrease in MABP ($P < 0.01$) (Table II). At 24 h, the pressure lowering effect of TNF-α was no longer present in any of the examined melanoma lines (Table I).

Data from the arterial blood gas analysis are shown in Table II. Plasma pH, $pCO_2$ and $[HCO_3^-]$ were significantly decreased in tumours 5 h after TNF-α injection. No changes were found in $pO_2$ or respiration rate at any of the examined time points.

### Discussion

The movement of molecules across vessel walls and in the interstitial matrix occurs by diffusion and convection. Convection results from a pressure gradient between the blood vessels and the tumour cells. Transport of low molecular weight substances is diffusion dominated, and delivery of small molecules can probably be increased by making the blood flow more uniform (Jain, 1994). Convection becomes important at higher molecular weights (e.g. albumin-bound drugs, antibodies or genes (Jain, 1994) and depends mainly on the pressure gradients between the vascular and interstitial space and the hydraulic conductivity (K). The equilibration of microvascular and interstitial pressures in tumours reduces the movement of large molecules by convection (Boucher and Jain, 1992).

The present study is the first to investigate the relationship between TNF-α and tumour interstitial fluid pressure in human melanoma xenografts. Melanomas are particularly interesting in this context, since clinical studies have shown impressive effects of the combination of TNF-α with melphalan and interferon-γ in locally perfused melanomas (Fraker and Alexander, 1993; Vaglini *et al.*, 1994). The mean IFP of untreated tumours was 14 mmHg, which is directly comparable with previously obtained clinical melanoma data (mean IFP = 14.3) (Boucher *et al.*, 1991). Other investigators have studied IFP in human melanoma xenografts and have found comparable baseline IFP values with no correlation between IFP and tumour size (Curti *et al.*, 1993; Tufto and Rofstad, 1995). In one clinical study, however, IFP increased with melanoma size (Boucher *et al.*, 1991).

TNF-α decreased IFP significantly in all of the three examined tumour lines 1–5 h after TNF-α injection (Table I). The animals in the 1 h experiment were treated differently from the animals in which the IFP was measured 5 and 24 h after TNF-α treatment, since the former animals were anaesthetised and the IFP measurement was already initiated when the tail vein injection was given. In the 5 and 24 h experiments the animals were restrained and injected 5 or 24 h before the IFP measurement. It might seem as if the IFP increases between the 1 h and 5 h control groups, but there is significant overlap between the data and no significant difference between the pretreatment and 5 h IFP values in any of the control groups. A general relationship between MABP and IFP has previously been demonstrated (Zlotecki *et al.*, 1993, 1995), and the microvascular pressure has been found to be the main driving force of IFP in solid tumours (Boucher and Jain, 1992). An increase in drug delivery requires an increase in transvascular pressure gradient and not only a drop in IFP (Netti *et al.*, 1995). The present decrease in MABP

**Table I** IFP measurements during the first 60 min after tail vein injection of TNF-α (500 μg kg$^{-1}$) and at 5 and 24 h after TNF-α injection

| | *MeWo* | | *P-MEL* | | *S-MEL* | |
|---|---|---|---|---|---|---|
| *IFP (mmHg)* | *Sodium chloride* | *TNF-α* | *Sodium chloride* | *TNF-α* | *Sodium chloride* | *TNF-α* |
| Pretreatment | 11.5<br>(6)<br>*n*=8 | 12<br>(5)<br>*n*=5 | 14.5<br>(4.5)<br>*n*=4 | 14.5<br>(9.5)<br>*n*=6 | 17<br>(3.5)<br>*n*=6 | 15<br>(6.5)<br>*n*=7 |
| 30 min | 14<br>(6)<br>*n*=8 | 14.5<br>(7.5)<br>*n*=5 | 13<br>(4.5)<br>*n*=4 | 16<br>(10)<br>*n*=6 | 18.5<br>(4)<br>*n*=5 | 13<br>(6.5)<br>*n*=7 |
| 60 min | 14.5<br>(7.5)<br>*n*=5 | 12<br>(7)<br>*n*=5 | 12<br>(5.5)<br>*n*=3 | 13.5<br>(7.5)<br>*n*=6 | 15.5<br>(4)<br>*n*=3 | 12.5<br>(4.5)<br>*n*=4 |
| 5 h | 18.5<br>(7.5)<br>*n*=6 | 12*<br>(2.5)<br>*n*=6 | 21.5<br>(6.5)<br>*n*=6 | 13.5*<br>(4.5)<br>*n*=6 | 21.5<br>(4.5)<br>*n*=6 | 11.5**<br>(3.5)<br>*n*=7 |
| 24 h | 14.5<br>(4)<br>*n*=9 | 11.5<br>(7)<br>*n*=7 | 16<br>(4.5)<br>*n*=6 | 12<br>(6)<br>*n*=6 | 15.5<br>(7)<br>*n*=7 | 12.5<br>(6)<br>*n*=8 |

Fluid communication could not be maintained in all tumours during the 60 min experiment, explaining the decreasing number of measurements during time. Numbers in parentheses are standard deviations. *$P < 0.05$. **$P < 0.01$.

**Table II** Mean arterial blood pressure, respiration rate and blood gas analysis performed 5 and 24 h after injection of TNF-α (500 μg $kg^{-1}$) or sodium chloride (controls)

| | *5 h* | | *24 h* | |
|---|---|---|---|---|
| | *Sodium chloride* | *TNF-α* | *Sodium chloride* | *TNF-α* |
| MABP (mmHg) | 111.1 | 78.6** | 100.4 | 106.3 |
| | (14.8) | (22.9) | (19.9) | (17.3) |
| | *n* = 18 | *n* = 20 | *n* = 23 | *n* = 19 |
| Respiration rate | 88 | 80 | 98 | 95 |
| | (22) | (20) | (21) | (18) |
| Blood gas analysis | | | | |
| pH | 7.15 | 7.07* | 7.19 | 7.18 |
| | (0.04) | (0.08) | (0.06) | (0.09) |
| $pCO_2$ (mmHg) | 58.8 | 51.6* | 52.2 | 51.8 |
| | (4.8) | (5.9) | (6.8) | (9.8) |
| $pO_2$ (mmHg) | 79.1 | 81.8 | 61.8 | 71.1 |
| | (15.6) | (17.2) | (9.7) | (18.3) |
| $[HCO_3^-]$ | 19.6 | 14.4** | 19.5 | 19.0 |
| | (2.2) | (2.8) | (3.3) | (3.4) |

Numbers in parentheses are standard deviations. *$P<0.05$. **$P<0.01$.

represents the main mechanism for the IFP reduction, but an actual increase in the pressure gradient across the vessel wall cannot be excluded by the present data. Thus, the previously demonstrated increase in antibody uptake 1–4 h after i.v. TNF-α injection (Folli *et al.*, 1993; Rowlinson-Busza *et al.*, 1995) might still be explained by an increase in vascular permeability surface area product and/or convection across the microvascular wall.

Both groups (TNF-α-treated and controls) were acidotic, presumably owing to the anaesthesia and the dorsal positon of the mouse during the cannulation surgery, which reduces the respiration rate of the animals (Y Boucher, unpublished observation). The further decrease in plasma pH 5 h after TNF-α injection compared with controls was accompanied by a decrease in bicarbonate concentration and $pCO_2$, indicating a partially compensated metabolic acidosis, as has been described previously in relation to hypotension after systemic TNF-α treatment (Kettelhut *et al.*, 1987).

**Acknowledgements**

CA Kristensen and M Nozue contributed equally to this work. The authors thank Ms Melody A Swartz and Drs Paolo A Netti and Fan Yuan for helpful comments on this manuscript. Supported by grant CA-56591 from the National Cancer Institute. CA Kristensen is a post doctoral fellow of The Michaelsen Foundation and The Danish Medical Research Foundation, Denmark.

### References

AMIKURA K, YEO K-T, DVORAK HF, ALEXANDER HR AND FRAKER DL. (1995). Expression of vascular endothelial growth factor correlates with tumor necrosis factor cytotoxicity *in vivo*. (abstract). *Proc. Am. Assoc. Cancer Res.*, **36**, 468.

ASHER A, MULÉ JJ, REICHERT CM, SHILONI E AND ROSENBERG SA. (1987). Studies on the anti-tumour efficacy of systemically administered recombinant tumor necrosis factor against several murine tumors *in vivo*. *J. Immunol.*, **138**, 963–974.

BAUDRY N AND VICAUT E. (1993). Role of nitric oxide in effects of tumor necrosis factor-α on microcirculation in rat. *J. Appl. Physiol.*, **75**, 2392–2399.

BOUCHER Y AND JAIN RK. (1992). Microvascular pressure is the principal driving force for interstitial hypertension in solid tumors: implications for vascular collapse. *Cancer Res.*, **52**, 5110–5114.

BOUCHER Y, KIRKWOOD JM, OPACIC D, DESANTIS M AND JAIN RK. (1991). Interstitial hypertension in superficial metastatic melanomas in humans. *Cancer Res.*, **51**, 6691–9994.

CURTI BD, URBA WJ, ALVORD WG, JANIK JE, SMITH II JW, MADARA K AND LONGO DL. (1993). Interstitial pressure of subcutaneous nodules in melanoma and lymphoma patients: changes during treatment. *Cancer Res.*, **53**, 2204–2207.

FOLLI S, PÉLEGRIN A, CHALANDON Y, YAO X, BUCHEGGER F, LIENARD D, LEJEUNE F AND MACH J-P. (1993). Tumor necrosis factor can enhance radio-antibody uptake in human colon carcinoma xenografts by increasing vascular permeability. *Int. J. Cancer*, **53**, 829–836.

FRAKER DL AND ALEXANDER HR. (1993). The use of tumor necrosis factor in isolated limb perfusions for melanoma and sarcoma. *Prin. Practice Oncol.*, **7**, 1–10.

JAIN RK. (1994). Transport phenomena in tumors. *Adv. Chem. Eng.*, **19**, 129–200.

KETTELHUT IC, FIERS W AND GOLDBERG AL. (1987). The toxic effects of tumor necrosis factor *in vivo* and their prevention by cyclooxygenase inhibitors. *Proc. Natl Acad. Sci. USA*, **84**, 4273–4277.

KILBOURN RG, GROSS SS, JUBRAN A, ADAMS J, GRIFFITH OW, LEVI R AND LODATO RF. (1990). $N^G$-methyl-L-arginine inhibits tumor necrosis factor-induced hypotension: implications for the involvement of nitric oxide. *Proc. Natl Acad. Sci. USA*, **87**, 3629–3632.

KRISTJANSEN PEG, BOUCHER Y AND JAIN RK. (1993). Dexamethasone reduces the interstitial fluid pressure in a human colon adenocarcinoma xenograft. *Cancer Res.*, **53**, 4764–4766.

LIENARD D, EWALENKO P, DELMOTTE J-J, RENARD N AND LEJEUNE FJ. (1992). High-dose recombinant tumor necrosis factor alpha in combination with interferon gamma and melphalan in isolation perfusion of the limbs for melanoma and sarcoma. *J. Clin. Oncol.*, **10**, 52–60.

NETTI PA, BAXTER LT, BOUCHER Y, SKALAK R AND JAIN RK. (1995). Time-dependent behaviour of interstitial fluid pressure inside tumors: implications for drug delivery. *Cancer Res.*, **55**, 5451–5458.

ROWLINSON-BUSZA G, MARAVEYAS A AND EPENETOS AA. (1995). Effect of tumour necrosis factor on the uptake of specific and control monoclonal antibodies in a human tumour xenograft model. *Br. J. Cancer*, **71**, 660–665.

TRACEY KJ, FONG Y, HESSE DG, MANOGUE KR, LEE AT, KUO GC, LOWRY SF AND CERAMI A. (1987). Anti-cachectin/TNF monoclonal antibodies prevent septic shock during lethal bacteraemia. *Nature*, **330**, 662–664.

TUFTO I AND ROFSTAD EK. (1995). Interstitial fluid pressure in human melanoma xenografts. Relationship to fractional tumor water content, tumor size, and tumor volume-doubling time. *Acta Oncol.*, **34,** 361–365.

VAGLINI M, BELLI F, AMMATUNA M, INGLESE MG, MANZI R, PRADA A, PERSIANI L, SANTINAMI M, SANTORO N AND CASCINELLI N. (1994). Treatment of primary or relapsing limb cancer by isolation perfusion with high-dose alpha-tumor necrosis factor, gamma-interferon, and melphalan. *Cancer*, **73,** 483–492.

ZLOTECKI RA, BOUCHER Y, LEE I, BAXTER LT AND JAIN RK. (1993). Effect of angiotensin II induced hypertension on tumor blood flow and interstitial fluid pressure. *Cancer Res.*, **53,** 2466–2468.

ZLOTECKI RA, BAXTER LT, BOUCHER Y AND JAIN RK. (1995). Pharmacologic modification of tumor blood flow and interstitial fluid pressure in a human tumor xenograft: network analysis and mechanistic interpretation. *Microvasc. Res.*, **50,** 429–443.